# Being red, blue and green: the genetic basis of coloration differences in the strawberry poison frog (*Oophaga pumilio*)

**DOI:** 10.1186/s12864-020-6719-5

**Published:** 2020-04-15

**Authors:** Ariel Rodríguez, Nicholas I. Mundy, Roberto Ibáñez, Heike Pröhl

**Affiliations:** 1 Institute of Zoology, University of Veterinary Medicine of Hannover, Bünteweg 17, 30559 Hannover, Germany; 2 Department of Zoology, University of Cambridge, Downing St, Cambridge, CB2 3EJ England; 3 Smithsonian Tropical Research Institute, Apartado Postal, 0843-03092 Panamá, República de Panamá; 4 Sistema Nacional de Investigación, Secretaría Nacional de Ciencia, Tecnología e Innovación, Apartado, 0816-02852 Panamá, República de Panamá

**Keywords:** Coloration genetics, Pigments, Gene expression, SNPs, Poison frog

## Abstract

**Background:**

Animal coloration is usually an adaptive attribute, under strong local selection pressures and often diversified among species or populations. The strawberry poison frog (*Oophaga pumilio*) shows an impressive array of color morphs across its distribution in Central America. Here we quantify gene expression and genetic variation to identify candidate genes involved in generating divergence in coloration between populations of red, green and blue *O. pumilio* from the Bocas del Toro archipelago in Panama.

**Results:**

We generated a high quality non-redundant reference transcriptome by mapping the products of genome-guided and de novo transcriptome assemblies onto a re-scaffolded draft genome of *O. pumilio*. We then measured gene expression in individuals of the three color phenotypes and identified color-associated candidate genes by comparing differential expression results against a list of a priori gene sets for five different functional categories of coloration – pteridine synthesis, carotenoid synthesis, melanin synthesis, iridophore pathways (structural coloration), and chromatophore development. We found 68 candidate coloration loci with significant expression differences among the color phenotypes. Notable upregulated examples include pteridine synthesis genes *spr*, *xdh* and *pts* (in red and green frogs); carotenoid metabolism genes *bco2* (in blue frogs), *scarb1* (in red frogs), and guanine metabolism gene *psat1* (in blue frogs). We detected significantly higher expression of the pteridine synthesis gene set in red and green frogs versus blue frogs. In addition to gene expression differences, we identified 370 outlier SNPs on 162 annotated genes showing signatures of diversifying selection, including eight pigmentation-associated genes.

**Conclusions:**

Gene expression in the skin of the three populations of frogs with differing coloration is highly divergent. The strong signal of differential expression in pteridine genes is consistent with a major role of these genes in generating the coloration differences among the three morphs. However, the finding of differentially expressed genes across pathways and functional categories suggests that multiple mechanisms are responsible for the coloration differences, likely involving both pigmentary and structural coloration. In addition to regulatory differences, we found potential evidence of differential selection acting at the protein sequence level in several color-associated loci, which could contribute to the color polymorphism.

## Background

Animal coloration plays important roles in intra- and interspecific communication, thermoregulation, predator avoidance and other ecological interactions with direct impact on individual fitness. Color phenotypes are often under strong local selection pressures and can be strikingly different among related species or populations [1–3]. For its functional significance, diversity, and the relative ease of obtaining comparable measurements, coloration is one of the most tractable traits in evolutionary research [4, 5].

The extended color palette exhibited by animals is produced by a combination of the selective absorption of light by different types of pigments and light scattering on reflective structures such as purine crystals or keratin [6–8]. Until very recently, knowledge of the genetic basis of vertebrate coloration has focused on a few species of mammals, birds and fish and strongly biased towards melanin-based coloration [9]. This situation is rapidly changing with the increase in power and affordability of genomic sequencing technologies.

In amphibians, reptiles and fish, integumentary coloration is produced by three main types of chromatophore cell: melanophores, xanthophores and iridophores [10]. Melanophores synthesize brown/black melanin pigment, xanthophores express yellow to red pteridine and/or carotenoid pigments, and iridophores produce reflective guanine crystals contributing to structural coloration. The typical arrangement of these cells is in a three-layered sandwich, with xanthophores overlying iridophores, and melanophores in the basal position, forming a “dermal chromatophore unit” [11].

Across vertebrates, the genetics of melanin-based coloration is relatively well studied and multiple genes involved in natural variation in melanin coloration have been identified in case studies on mammals, reptiles, birds and fishes [5]. In contrast, comparatively, little is known about the genetic basis of carotenoid and pteridine based pigmentation. The biosynthetic pathway for pteridine synthesis, based on guanosine triphosphate (GTP), was elucidated in zebrafish [12], and later generalized for vertebrates [13]. Unlike melanin and pteridines, carotenoids cannot be produced de novo by vertebrates but have to be obtained by ingestion, and hence their availability is environmentally-dependent [14]. Assimilation, modification and accumulation of carotenoids in their target tissues involve numerous steps and results in a large number of molecular interactions impacting many aspects of vertebrate physiology [15, 16]. Variation in guanine-based structural coloration produced by iridophores is poorly studied, with most work being performed in zebrafish [14].

In contrast to fish, birds and mammals little is known about the molecular and genetic basis of coloration in amphibians. One of the most remarkable examples of natural intra-specific polymorphism in amphibians is the small and visually conspicuous strawberry poison frog *Oophaga pumilio* (Schmidt, 1857), a member of Dendrobatidae that inhabits tropical rain forests in Central America. While the ancestral and most frequent color phenotype is bright red [17, 18], a broad array of color morphs have evolved on the mainland and especially the islands of the Bocas del Toro archipelago in Panama [19]. The dorsal coloration of these frogs is considered aposematic since alkaloids from their insect prey are sequestered in skin glands as chemical protection to discourage predators such as birds [20–22]. Multiple scenarios have been proposed to explain the staggering diversity of color phenotypes in this frog. Summers et al. [23] compared the phylogeography of the color polymorphic *O. pumilio* with two sympatric, color monomorphic species and inferred that sexual selection was involved in driving the rapid divergence in color and pattern between populations of *O. pumilio.* In this species, females make an important parental care investment and are extremely choosy with their mates displaying a significant preference for brightly colored males of their own color morph [24–28]. Maan and Cummings [29] demonstrated that color diversity in *O. pumilio* is also tightly linked to variation in toxicity and proposed that the polymorphism observed in Bocas del Toro might derive from an interaction between environmental heterogeneity of alkaloid availability, varying predation pressure and sexual selection by females. On the other hand, coalescent simulations suggest that, due to recent population expansions and the small island population sizes, genetic drift might have played a major role in the diversification of color across populations [30]. More recently, Yang et al. [31] analyzed female attraction and male aggression experiments in a cross-fostering study and found a combination of rival and sexual imprinting in these frogs, which could reduce gene flow between individuals that bear divergent mating traits and set the stage for speciation by sexual selection.

While the ecological and evolutionary factors contributing to the fascinating color divergence in *Oophaga pumilio* populations have been investigated from different angles, the contribution of molecular processes has been neglected so far. Breeding experiments show that offspring of crosses between color phenotypes typically display a mixture of parental coloration but with color pattern if one parent showed color pattern, which is suggestive of a single locus control of color pattern and a polygenic control of coloration [32]. A recent study on *Dendrobates auratus*, another species of Dendrobatidae, identified a large number of differentially expressed genes likely responsible for coloration differences, some of which showed single-nucleotide polymorphism (SNP) variation between color morphs [33], lending support to the polygenic control hypothesis.

In order to obtain a molecular perspective on the genomic basis of color polymorphism in amphibians, we herein studied three plain colored and strikingly divergent morphs of *O. pumilio* showing blue, red and green dorsal skin color. We used a combination of methods for gene expression quantification and single-nucleotide polymorphism (SNP) detection using RNA-seq data obtained from dorsal skin of wild-captured animals to identify candidate genes related to color variation. We hypothesized that red and green frogs would have up-regulated pteridine and/or carotenoid pathways, whereas blue frogs would have upregulated iridophore and/or melanogenic pathways involved in structural coloration.

## Results

### Draft genome re-scaffolding

The published *Oophaga pumilio* reference genome is a heavily fragmented draft, containing 7,182,834 scaffolds with N50 = 79,909 and largest contig (LC) = 0.9 Mb. Re-scaffolding of this draft with paired RNA-Seq reads in P_RNA software resulted in substantial improvements in contiguity (631,034 scaffolds, N50 = 116,040, LC = 1.7 Mb). This re-scaffolded genome had an improved BUSCO score (85.5% of completeness vs the original 76.6%) and was therefore used for subsequent analyses. Statistics of the two assemblies are provided in SMTable 1.

### Reference transcriptome assembly and annotation

The genome-guided and de novo assemblies resulted in 1,080,547 (N50 = 876) and 980,876 (N50 = 1024) transcripts respectively. A large fraction of these transcripts (2,031,063; 98.5%) aligned to the re-scaffolded draft genome and were combined in a non-redundant and comprehensive PASA transcriptome including 903,736 transcript sequences derived from 617,432 PASA clusters in the genome (representing gene structures from transcriptionally active regions which we tentatively assume as genes). The BUSCO scoring of this reference transcriptome indicated 94.5% completeness, 3.0% fragmentation, and 2.5% missing genes of the 2586 vertebrate bench-marking genes. This reference transcriptome includes 274,940 ORF-containing transcripts, 161,968 of which had positive blast hits against the UniProt database. Of these, 92,442 transcripts were identified as UniProt orthologs and 69,526 as paralogs. The coding transcripts originate from 35,953 distinct PASA clusters (genes) in the reference genome including 12,821 orthologs to sequences in UniProt database and 23,132 paralogs. Subsequent functional interpretations of results were restricted to the subset of orthologous coding genes.

### Differential expression analyses

**Fig. 1:**
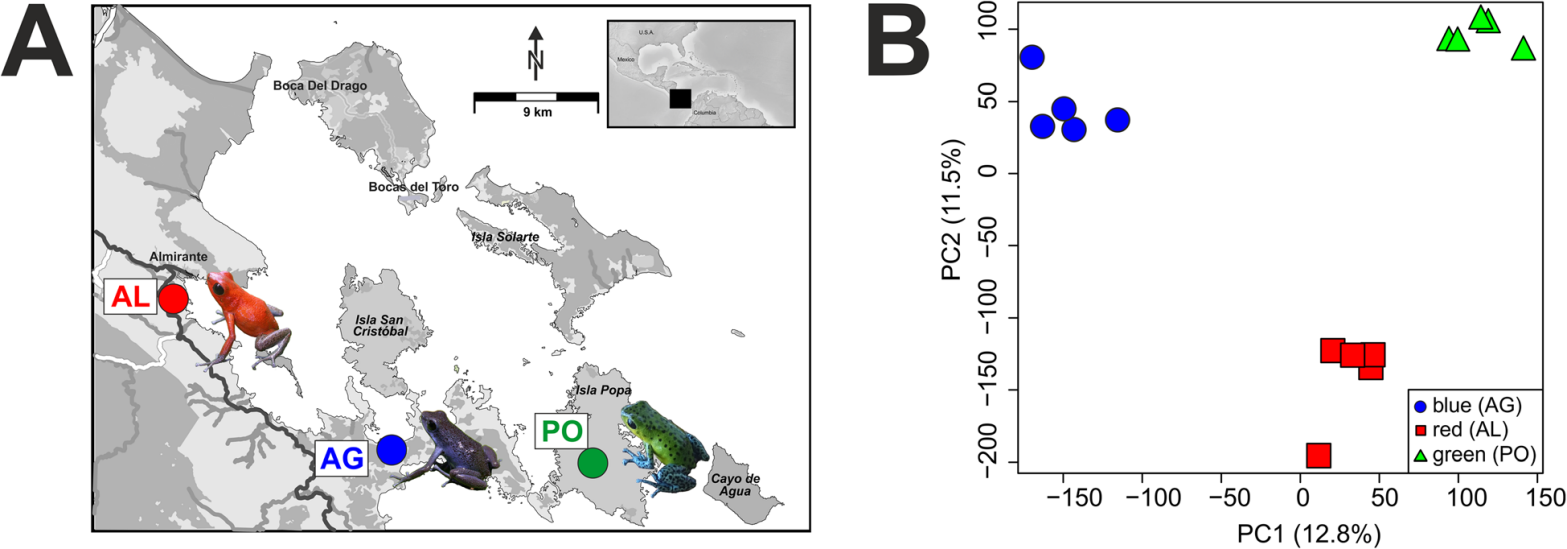
Sampling scheme and general gene expression patterns. A) Geographic location of localities in the Bocas del Toro archipelago where *Oophaga pumilio* samples were obtained (AL, Almirante; AG, Aguacate; PO, Popa) and their associated color phenotypes (inset photos). B) Plot of the principal component analysis summarizing the expression pattern across samples of the three color phenotypes. The background map in A (© OpenStreetMap contributors) was created with open data cartography licensed under a Creative Commons Attribution-ShareAlike 2.0 license (CC BY-SA, https://www.openstreetmap.org/copyright)

**Fig. 2:**
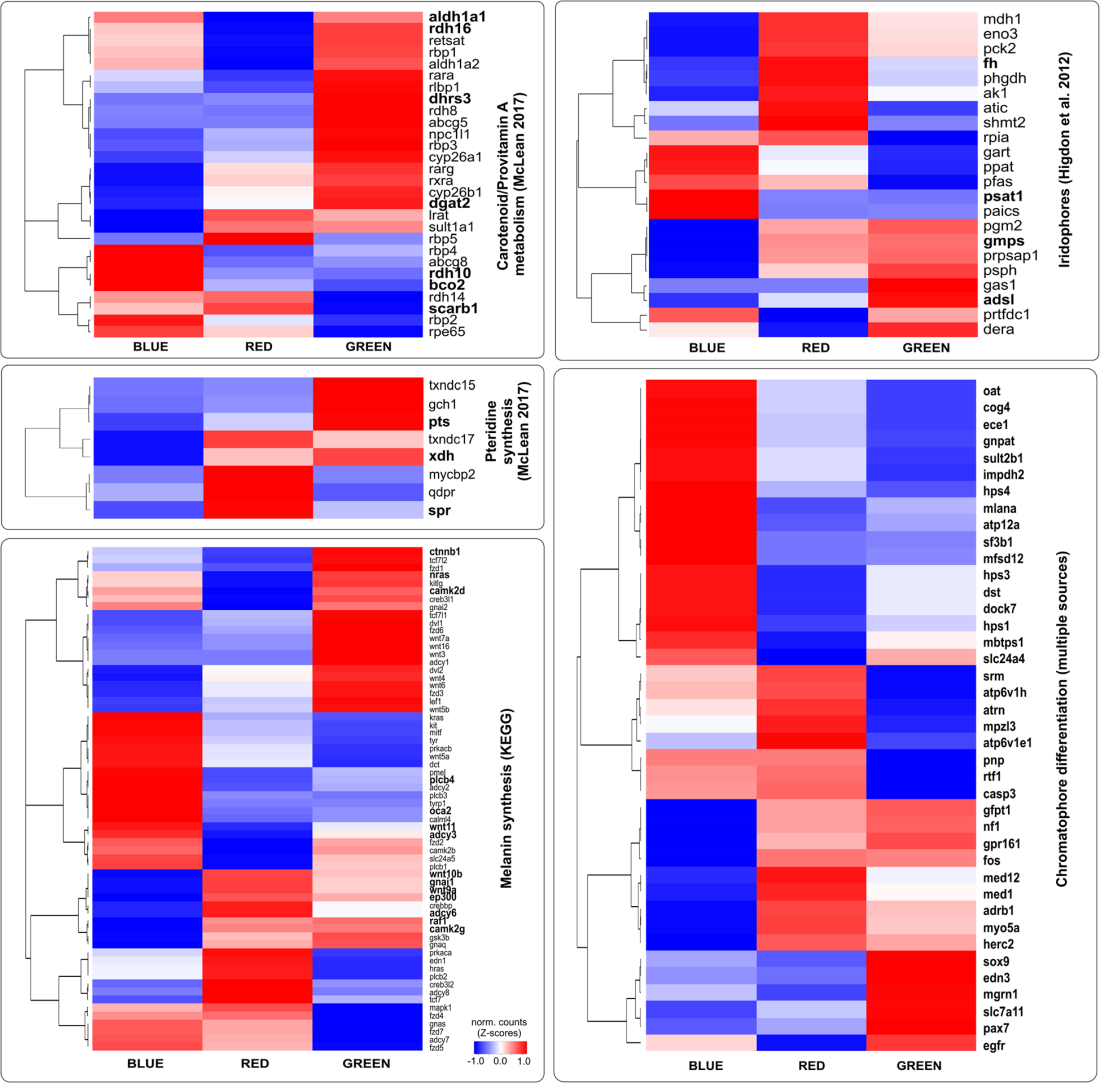
Expression patterns of genes in three color phenotypes of *Oophaga pumilio* classified into five functional groups of color-associated genes (pigment synthesis pathways, guanine synthesis in iridophores, and chromatophore differentiation). Each heat map plot was simplified by averaging expression values across all samples in each color morph to show the expression profiles of all genes in each group. DE genes are highlighted in bold except for the chromatophore differentiation where only DE genes are shown. Details of the gene sets are presented on the main text and SM

A total of 24,390 coding genes were expressed in the *O. pumilio* skin samples. A PCA analysis on expression levels in the fifteen samples showed strong clustering within populations and divergence between populations (Fig. 1). *Sleuth* analyses identified 2639 differentially expressed (DE) genes between the three color morphs (SMTable 2). Of these, 1445 were orthologs to Uniprot genes and the inspection of the expression profiles identified six DE gene clusters with functions related to angiogenesis, the gonadotropin-releasing hormone receptor, and multiple signaling pathways (SMFigure 1, SMTable 3). We identified 68 DE genes linked to pigment production, structural coloration in iridophores, and pigment-cell differentiation in previous studies (Table 1.). Seven genes were DE in the carotenoid metabolism pathway; *rdh10* and *bco2* were up-regulated in blue frogs, while *dgat2* and *dhrs3* were up-regulated in green frogs. In red frogs the *aldh1a1* enzyme was down-regulated while *scarb1* was up-regulated in comparison to blue and green frogs (Table 1, Fig. 2). Three DE genes were found in the pteridine synthesis pathway: *spr* was upregulated in red frogs, *pts* was up-regulated in green frogs, *xdh* was up-regulated in red and green frogs, and no gene was up-regulated in blue frogs (Table 1, Fig. 2). Fourteen DE genes were found in the melanin synthesis pathway: *oca2* and *plcb4* were up-regulated in blue frogs; the *ctnnb1* gene was up-regulated in green frogs while *wnt10b*, *gnai1*, *wnt9a*, *ep300*, *adcy6*, *raf1* and *camk2g* were up-regulated in green and red frogs (Table 1, Fig. 2). Four DE genes were found in the iridophore guanine synthesis pathway: *psat1* was upregulated in blue frogs, *adsl* was up-regulated in green frogs, and *fh* was up-regulated in red frogs. Additionally, we found 40 DE genes previously linked to chromatophore development and differentiation (Table 1, Fig. 2). Interesting candidates were *pax7*, and *sox9*, which were upregulated in green frogs; *med1*, *med12*, and *myo5a*, which were upregulated in red frogs; and *dock7*, *hps1*, *hps3*, *hps4* and *sf3b1* which were up-regulated in blue frogs.

**Table 1:** Differentially expressed genes between blue, green and red color phenotypes of *Oophaga pumilio* previously linked to coloration

rank	gene	transcripts	q-val	expression-pattern	pigmentation role
88	*dgat2*	4	0.000	GREEN > RED = BLUE	carotenoid metabolism
102	*rdh16*	4	0.000	BLUE = GREEN > RED	carotenoid metabolism
145	*scarb1*	1	0.000	RED > BLUE > GREEN	carotenoid metabolism
371	*rdh10*	2	0.000	BLUE > RED = GREEN	carotenoid metabolism
691	*bco2*	6	0.002	BLUE > RED = GREEN	carotenoid metabolism
969	*dhrs3*	1	0.005	GREEN > RED = BLUE	carotenoid metabolism
1124	*aldh1a1*	7	0.008	BLUE = GREEN > RED	carotenoid metabolism
240	*adsl*	8	0.000	GREEN > RED = BLUE	guanine synthesis in iridophores
525	*psat1*	4	0.001	BLUE > RED = GREEN	guanine synthesis in iridophores
1807	*gmps*	7	0.024	RED = GREEN > BLUE	guanine synthesis in iridophores
1871	*fh*	5	0.025	RED > GREEN > BLUE	guanine synthesis in iridophores
12	*camk2g*	6	0.000	RED = GREEN > BLUE	melanin synthesis
91	*gnai1*	7	0.000	RED > GREEN > BLUE	melanin synthesis
98	*ctnnb1*	13	0.000	GREEN > RED = BLUE	melanin synthesis
197	*raf1*	8	0.000	RED = GREEN > BLUE	melanin synthesis
481	*wnt9a*	3	0.001	RED > GREEN > BLUE	melanin synthesis
521	*adcy6*	4	0.001	RED > GREEN > BLUE	melanin synthesis
771	*wnt11*	3	0.003	BLUE > GREEN > RED	melanin synthesis
797	*nras*	3	0.003	BLUE = GREEN > RED	melanin synthesis
1718	*camk2d*	4	0.022	BLUE = GREEN > RED	melanin synthesis
1939	*ep300*	4	0.027	RED > GREEN > BLUE	melanin synthesis
2036	*plcb4*	9	0.031	BLUE > RED = GREEN	melanin synthesis
2100	*wnt10b*	6	0.032	RED > GREEN > BLUE	melanin synthesis
2571	*oca2*	3	0.048	BLUE > RED = GREEN	melanin synthesis
2620	*adcy3*	2	0.050	BLUE > GREEN > RED	melanin synthesis
119	*xdh*	9	0.000	RED = GREEN > BLUE	pteridine synthesis
853	*spr*	2	0.004	RED > GREEN > BLUE	pteridine synthesis
975	*pts*	2	0.005	GREEN > RED = BLUE	pteridine synthesis
1282	*sox9*	4	0.010	GREEN > RED = BLUE	chromatophore differentiation
167	*atp12a*	10	0.000	BLUE > RED = GREEN	chromatophore differentiation
280	*hps3*	14	0.000	BLUE > GREEN > RED	chromatophore differentiation
1509	*hps4*	5	0.016	BLUE > RED = GREEN	chromatophore differentiation
2260	*hps1*	8	0.037	BLUE > GREEN > RED	chromatophore differentiation
2176	*myo5a*	2	0.034	RED > GREEN > BLUE	chromatophore differentiation
1180	*slc7a11*	2	0.009	GREEN > RED = BLUE	chromatophore differentiation
316	*med1*	7	0.000	RED > GREEN > BLUE	chromatophore differentiation
32	*egfr*	7	0.000	BLUE = GREEN > RED	chromatophore differentiation
55	*sult2b1*	5	0.000	BLUE > RED = GREEN	chromatophore differentiation
128	*nf1*	19	0.000	RED = GREEN > BLUE	chromatophore differentiation
327	*gpr161*	5	0.000	RED = GREEN > BLUE	chromatophore differentiation
337	*fos*	4	0.000	RED = GREEN > BLUE	chromatophore differentiation
453	*gnpat*	10	0.001	BLUE > RED = GREEN	chromatophore differentiation
457	*mpzl3*	1	0.001	RED > BLUE > GREEN	chromatophore differentiation
487	*srm*	5	0.001	RED > BLUE > GREEN	chromatophore differentiation
491	*atp6v1h*	3	0.001	RED > BLUE > GREEN	chromatophore differentiation
572	*mfsd12*	5	0.001	BLUE > RED = GREEN	chromatophore differentiation
611	*cog4*	14	0.001	BLUE > RED = GREEN	chromatophore differentiation
641	*dst*	11	0.002	BLUE > GREEN > RED	chromatophore differentiation
666	*oat*	4	0.002	BLUE > RED = GREEN	chromatophore differentiation
722	*slc24a4*	5	0.002	BLUE = GREEN > RED	chromatophore differentiation
813	*mbtps1*	6	0.003	BLUE > GREEN > RED	chromatophore differentiation
1022	*mlana*	5	0.006	BLUE > RED = GREEN	chromatophore differentiation
1053	*herc2*	13	0.006	RED > GREEN > BLUE	chromatophore differentiation
1130	*adrb1*	4	0.008	RED > GREEN > BLUE	chromatophore differentiation
1270	*casp3*	3	0.010	BLUE = RED > GREEN	chromatophore differentiation
1271	*dock7*	2	0.010	BLUE > GREEN > RED	chromatophore differentiation
1388	*atrn*	4	0.013	RED > BLUE > GREEN	chromatophore differentiation
1457	*ece1*	6	0.015	BLUE > RED = GREEN	chromatophore differentiation
2086	*mgrn1*	3	0.032	GREEN > RED = BLUE	chromatophore differentiation
2118	*med12*	6	0.033	RED > GREEN > BLUE	chromatophore differentiation
2141	*atp6v1e1*	3	0.033	RED > BLUE > GREEN	chromatophore differentiation
2438	*edn3*	5	0.043	GREEN > RED = BLUE	chromatophore differentiation
2439	*gfpt1*	4	0.043	RED = GREEN > BLUE	chromatophore differentiation
2468	*pnp*	3	0.044	BLUE = RED > GREEN	chromatophore differentiation
2500	*sf3b1*	6	0.045	BLUE > RED = GREEN	chromatophore differentiation
2592	*rtf1*	1	0.049	BLUE = RED > GREEN	chromatophore differentiation
2134	*impdh2*	5	0.033	BLUE > RED = GREEN	chromatophore differentiation
800	*pax7*	1	0.003	GREEN > RED = BLUE	chromatophore differentiation

For each gene, the number of transcripts compared, q-value, expression pattern and color-associated function are presented. The expression pattern represents the observed differences in mean transcript counts aggregated by gene and color morphs (see Fig. 2)

### Gene set enrichment and over-representation analyses

Significant enrichment was detected in the genes of the pteridine synthesis pathway in red vs blue and green vs blue comparisons. The remaining pigment-synthesis gene sets showed no significant enrichment in any of the pair-wise comparisons (Table 2). Results of the over-representation analysis of the entire set of 1445 DE genes indicated a total of 312 enriched gene ontology (GO) terms of different categories (biological process: 183, cellular component: 71, and molecular function: 58; SMTable 4). These include categories related to biological processes including: protein folding response, endoplasmic reticulum stress response, establishment or maintenance of cell polarity and amino acid and organonitrogen compound metabolic process. The gene ontology categories are localized in multiple cell compartments, from the cytoplasm to pigment granules, vesicles and the endoplasmic reticulum. Over-represented molecular functions included: RNA binding, oxidoreductase activity, protein binding, organic cyclic compound binding, small molecule binding, and chaperone binding. A graphical overview of the over-represented categories, as obtained from REVIGO, is presented in SMFigure 2.

**Table 2:** Results of the gene-set enrichment analysis (GSEA) testing for differences in expression of color-associated gene sets between color phenotypes

contrast	Pteridine synthesis	Carotenoid metabolism	Melanin synthesis	Guanine synthesis (Iridophore)
RED vs BLUE	**0.026**	0.521	0.618	0.698
GREEN vs BLUE	**0.032**	0.378	0.507	0.752
GREEN vs RED	0.844	1.000	0.510	0.635

For each pair-wise comparison the false detection ratio (FDR) is shown with significant results (FDR < 0.25) highlighted in bold. Genes included in each set and their expression profiles are illustrated in Fig. 2

### SNPs and signatures of selection

**Fig. 3:**
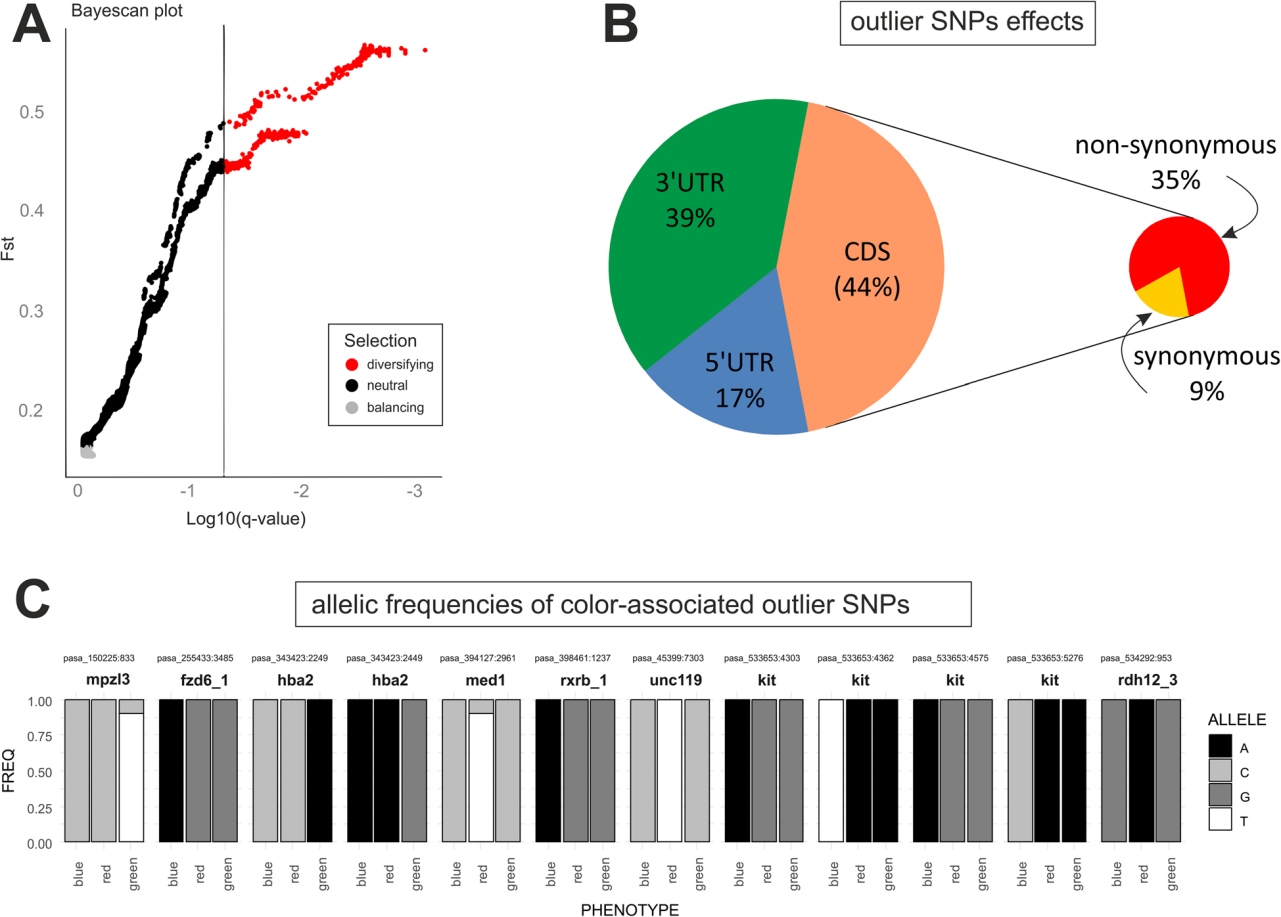
Signatures of selection on single-nucleotide polymorphisms (SNPs) in the transcriptome of *Oophaga pumilio*. A) Graphical results of the BayeScan analysis, the plot shows, for each of the 398,910 bi-allelic SNPs tested, the Fst between the three color phenotypes and their corresponding q-values (SNPs under diversifying selection, q < 0.05 and α > 0, are highlighted in red). B) Pie chart showing the positions and predicted effects of the 370 outlier SNPs detected in the BayeScan analysis. C) Allelic frequencies of the outlier SNPs occurring in color-associated genes, each bar chart shows the frequency of alleles in each population

A total of 1,917,067 SNPs were identified with the GATK pipeline in the assembled superTranscriptome of *O. pumilio*. Of these, 398,910 bi-allelic SNPs passed the sample and genotype coverage thresholds and were subjected to BayeScan analyses. Results identified a total of 370 outlier SNPs showing signature of directional selection with *F*_st_ among the three populations ranging between 0.44 and 0.57 with an upper trace in the *F*_st_ vs *q*-value plot representing the SNPs with maximal differentiation between color morphs – fixed (0 or 1) allelic frequencies (Fig. 3a). The outlier SNPs were located in 762 transcripts, with twice as many outlier SNPs in 3′-untranslated regions (3′-UTR, 562, 39%) compared to 5′ untranslated regions (5’UTR, 252, 17%). Among outlier SNPs in the coding sequence (CDS, 637, 44%) there were four times as many non-synonymous (512) compared to synonymous (127) variants (Fig. 3b). Outlier SNPs occurred on 162 annotated genes and 39% of these (65 genes) also showed significant expression differences which could represent functional SNPs under selection that affect gene expression (SMTable 5). Twelve of the outlier SNPs occurred on eight genes associated with pigmentation (Table 3) and, of these, *kit* showed the strongest signal, with four linked outlier SNPs, spanning ~ 630 bp, fixed in blue frogs (Fig. 3c). One of these SNPs represented a non-synonymous substitution while the other three lay in the 3’UTR region (SMFigure 3). It is worth noting that no significant differences in expression were detected for this gene in *Sleuth* tests (SMFigure 4).

**Table 3:** Color-associated genes with SNPs showing signatures of directional selection among the three color phenotypes of *O. pumilio* studied

SNP	alleles	q-value	alpha	***F***_**st**_	gene	Link to pigmentation
pasa_150225:833	C/T	0.042	1.396	0.447	mpzl3_2	severe skin and hair abnormalities in mice (p)
pasa_255433:3485	G/A	0.032	1.623	0.498	fzd6_1	melanin synthesis (p)
pasa_343423:2249	C/A	0.002	1.916	0.561	hba2	hyperpigmented human skin
pasa_343423:2449	A/G	0.002	1.940	0.567	hba2	hyperpigmented human skin
pasa_394127:2961	C/T	0.046	1.387	0.445	med1	retinal pigmentation
pasa_398461:1237	G/A	0.006	1.795	0.535	rxrb_1	carotenoid metabolism (p)
pasa_45399:7303	C/T	0.018	1.718	0.518	unc119	retinal pigmentation
pasa_533653:4303	G/A	0.002	1.935	0.566	kit	melanin synthesis
pasa_533653:4362	A/T	0.003	1.922	0.563	kit	melanin synthesis
pasa_533653:4575	G/A	0.002	1.919	0.562	kit	melanin synthesis
pasa_533653:5276	A/C	0.002	1.935	0.565	kit	melanin synthesis
pasa_534292:953	G/A	0.007	1.755	0.526	rdh12_3	carotenoid metabolism (p)

For each SNP entry, the position and alternative alleles, as well as BayeScan results (posterior probability, q-value, alpha, *F*_st_) are presented along with the gene symbol and potential link to pigmentation. Gene symbols followed by underscore and a number represent potential paralogs and their functions (p) are less reliable

## Discussion

Our results support a key role of regulatory control but also a potential role of single-nucleotide polymorphisms in shaping the observed differences in skin coloration in *O. pumilio* frogs of the Bocas del Toro archipelago. The multidimensional nature of the molecular basis for color diversity discovered in this study is not surprising, considering that skins of amphibians are multilayered, three-dimensional structures of several cell types that often contain multiple pigment types and structural features [34]. It is the interplay among the reflection and absorbance of light of different wavelengths with the presence and absence of certain pigments, aggregation or dispersion of pigment containing organelles (e.g. melanosomes) which determines the color phenotype of the animal [34, 35].

### Gene expression differences

A great diversity of pteridines can be found in amphibian skin, including *Oophaga*, and these contribute to yellow, orange or red pigmentation [10, 36]. Our results support an important role of pteridines in the pigmentation of red and green color morphs of *O. pumilio*. Importantly, the full set of eight pteridine synthesis genes were significantly more highly expressed in red versus blue and green versus blue frogs, the expected pattern if they are functionally involved in coloration differences. Of these eight, three were differentially expressed at the individual level (*spr*, *pts*, and *xdh*) and these have all been previously implicated in variation in red to yellow skin pigmentation in studies of fishes and reptiles [12, 13, 37, 38]. In particular, increased expression of *spr* has been related to the red morphs of the European wall lizard [37], and red frogs had the highest expression of this gene. Earlier studies on anurans identified *xdh* as a candidate for pigmentation variation in the tree frog *Agalychnis dacnicolor*, where *xdh* inhibition resulted in reduced pigmentation supporting the role of this enzyme in the synthesis of pterorhodin, a red pigment [39]. More recently, a gene expression study on captive bred *Dendrobates auratus*, a dendrobatid frog, reported that blue-black morph individuals show lower expression of *xdh* transcripts than the brown or greenish-blue dorsum morphs [33]. This matches perfectly with the expression profile observed in our study and supports the role of *xdh* in the synthesis of pteridines responsible for the red and green skin coloration in *O. pumilio*. *Pts* and *spr* regulate the second and third steps in the pteridine synthesis pathway while *xdh* regulates the final production of several yellow pteridine pigments [12].

Only a few genes have previously been directly implicated in carotenoid coloration and so it is notable that two of them were differentially expressed among *O. pumilio* populations. *Bco2* (Beta, beta-carotene 9,10-oxygenase) encodes a carotenoid cleaving enzyme which explains white to yellow variation in the coloration of chicken legs [40], and has also low expression in yellow wall lizards [11]. Here we found that *bco2* had higher expression in blue than green or red frogs, consistent with a potential role in cleaving carotenoids in the skin of blue frogs. The second locus, *scarb1*, is a carotenoid transporter involved in carotenoid uptake into cells, responsible for the white canary phenotype when its function is disrupted [41]. Again, consistent with this function, *scarb1* had highest expression in red frogs. Interestingly, other genes involved in carotenoid metabolism are more highly expressed in green and blue frogs than red frogs. However those genes that are significantly higher expressed in green or blue frogs (*dgat2*, *rhd10*, *rdh16*, *dhrs3* and *aldh1a1*) mostly code for enzymes of retinol metabolism which act downstream of carotenoid cleavage and are unlikely to affect carotenoid coloration [42]. We found no compelling evidence that a skin-expressed cytochrome P450 gene (CYP) is involved in production of ketocarotenoids in red frogs as occurs in red birds and turtles [43–45]. We found 30 CYP genes in the list of DE genes but except for the *cyp8b1* gene (involved in bile acid synthesis and not a good candidate), all of them were potential paralogs which complicates functional interpretations. A strikingly complex mixture of carotenoids has been recently identified in skins of the orange color morph of *O. pumilio* [46] and further studies will be required to elucidate the particularities of carotenoid metabolism in these frogs.

The structural blue coloration in frogs likely depends on both the iridophore layer and the underlying melanophore layer and so we expected that genes affecting blue coloration should be expressed in these cells. A strong candidate from iridophores is *psat1*, which has higher expression in blue than red or green frogs and is in the biosynthetic pathway for guanine. When comparing our findings in Fig. 2 (heatmap) to the guanine synthesis cycle for zebrafish [47] an interesting pattern emerges: those genes which have higher expression in blue frogs are those coding for enzymes that contribute to the production of SAICAR (phosphoribosylaminoimidazole-succinocarboxamide) from 5-Phosphoribosyl 1-Pyrophosphate (PRPP) (*ppat, pfas, gart, paics*; purine synthesis) and *prtfdc1* that directly produces guanine from guanosine monophosphate (GMP, i.e. guanine-specific), while the genes that are more highly expressed in red frogs are mainly located in the citrate cycle (*fh, mdh1a, pck2, eno3*) and serine/glycine pathway (*phgdh, shmt2*) which does not directly lead to the production of purine or guanine (except *atic*). There is no clear pattern for the location of the genes in the guanine synthesis cycle, which are more highly expressed in the green frogs. In summary, the blue frogs have genes upregulated that produce SAICAR, which is the starting product for purine and guanine synthesis, while red frogs have genes upregulated that recycle SAICAR for precursors of the citrate cycle, glycolysis and production of amino acids (serine, glycine). *Psat1* (highly expressed in blue frogs) reconnects the serine/glycine pathway back to purine synthesis.

For melanophores, two interesting candidates that might affect blue coloration in *O. pumilio* are *oca2* and *myo5a*. These genes show an opposed expression pattern in blue frogs, the *oca2* gene shows up-regulation while the *myo5a* gene is downregulated. *Oca2* plays an important role in melanogenesis, with mutations leading to paler coloration in fish and humans [48]. The *myo5a* gene encodes a protein involved in short-range movement of melanosomes along actin filaments and plays a role in moving melanosomes to the dendrites. Defects in *myo5a* lead to the lavender (pale grey) phenotype in many mammalian and avian species [e.g. in horses, 56], in which the transfer of melanosomes to keratinocytes occurs. In species such as frogs where this process does not occur, the phenotypic effects may be different.

Several of the differentially expressed genes involved in chromatophore development are also interesting candidates. Disruption of *sf3b1*, which is upregulated in blue frogs, is responsible for proliferation of dermal melanocytes and blue coloration via the Tyndall effect in humans[49].

Blue frogs also show increased expression of the *hps1*, *hps3*, *hps4* genes. These genes encode three Hermansky-Pudlak syndrome proteins, which in humans are linked to oculocutaneous albinism with abnormally light coloring of the skin, hair, and eyes. One of them (*hps3*) was also found up-regulated in all three color phenotypes of *Dendrobates auratus* that included blue skin patches [33]. The *pax7* and *sox9* genes are two interesting candidates that showed increased expression in green frogs and might be responsible for this particular coloration. The *pax7* gene plays a key role in the differentiation of xanthophore precursor cells and its absence results in a complete depletion of differentiated xanthophores in embryos as well as in adult zebrafish [50]. The expression of the *sox9* gene is known to stimulate melanin production and leads to increased human skin pigmentation in response to UV stimulus [51].

Comparison of our gene expression results with those of Stuckert et al. [33] revealed 240 genes showing differential expression between color morphs in both species (i.e. *O. pumilio* and *D. auratus*, SMTable 6). The list includes genes related to pteridine synthesis (*xdh*), melanogenesis (*gnai1*, *raf1*), 12 genes involved in melanophore genesis and differentiation (e.g. *atp12a, hps3*, and *sox9*). Although the color differences among the morphs differ in the two species, some of these genes may play general roles in amphibian coloration and should be the target of future studies.

### SNP variation

The results obtained at the SNP level suggest that sequence variation in multiple loci may be contributing to adaptive color variation among these frogs. The *kit* gene encodes a growth factor receptor protein which is known to play a key role regulating the melanin synthesis pathway as well as melanophore survival and positioning of kit receptor-expressing melanocytes [52, 53]. The existence of four variants of *kit* exclusive to blue frogs strongly suggest that this gene might have an important role in color determination in these frogs, a subject that should be scrutinized further with additional population surveys and laboratory studies. Interestingly, two other studies on species of the Dendrobatidae family have pointed to variants in another gene, the *mc1r* gene (encoding the melanocyte-stimulating hormone receptor) as a main candidate for explaining pigmentation differences [33, 54]. However, we did not find any variation in the *mc1r* gene among the color morphs studied, including the variant position in the *O. histrionica* species complex (SMFigure 5). In the two previous studies the phenotypic differences between the color morphs tested included color patterning variation, while in our study the differences are solely related to background coloration itself. This suggests that these variants in the *mc1r* gene might be involved in color patterning and not related to background coloration itself, which would match the profile of the candidate color-patterning gene inferred by the cross-breeding experiments of Summers et al. [32].

The abundance of outlier SNPs landing on 3′ untranslated regions (UTR) is noteworthy. Although such a pattern might arise by chance if the 3’UTRs in the dataset are longer than the 5’UTRs, inspection of feature length distribution in our reference transcriptome does not indicate that length differences could explain the abundance of SNPs in the 3’UTR regions (SMFigure 6). Polymorphisms in the 3’UTR can alter the secondary structure and affect mRNA stability [55] and these regions are also important targets of post-transcriptional regulation via miRNAs [56, 57].

The finding that a high proportion of outlier SNPs occurred in genes with differential expression (40%) gives high confidence that the BayeScan analysis has uncovered functionally relevant outliers. The selection test implemented in BayeScan accounts for uncertainty in allele frequencies providing unbiased estimates even with very small sample sizes, albeit with the risk of a low power [58]. Given the small number of individuals per population, the statistical power of our implementation of BayeScan is modest and it is likely that the set of outlier SNPs identified in this study represents only a fraction of the total SNPs experiencing positive selection. On the other hand, genomic selection scans are sensitive to the strength of selection and local recombination rate as well as the underlying genetic structure, demography and migration rate of the sampled populations [59]. Characterizing and accounting for those sources of variation should be the goal of future in-depth studies on the targets of selection in these frogs.

### Future directions

Our results on SNP variation and gene expression concern only those genes that are consistently expressed in the skin and hence represent a subset of all the genomic loci involved in skin pigmentation of these frogs. Additionally, SNP calling from RNA-Seq data cannot capture variation involved in untranscribed regulatory regions that may underlie the majority of adaptive phenotypic variation, unless linkage disequilibrium is high between regulatory and coding regions [60]. We are, however, confident that our results provide an extensive list of color-associated genes that represent good candidates for future insights into the genomics of color polymorphism in these frogs.

The limited genomic resources available for *O. pumilio* surely limit our interpretations and an adequate assembly and annotation of the genome will allow a more detailed interpretation of our results. Besides an adequate reference genome, subsequent studies should address the following goals: 1) the comparison of gene expression and SNPs among additional populations with blue, green and red dorsal coloration to find out whether similar coloration in geographically distinct localities rely on the same underlying molecular processes; 2) analyses of differential expression in additional organs that may be involved in pigment uptake and metabolism (e.g. liver and gut); 3) the inclusion of additional frog color morphs (yellow, orange, black-white) not considered in this research, and 4) the assessment of gene expression of candidate genes in hybrid populations; for example in a contact zone where red, blue and all shades of intermediate phenotypes (purple) co-occur (e.g. Yang et al. [61]).

## Conclusions

The three populations of frogs exhibiting red, blue and green coloration showed highly divergent gene expression in their skins. The strong signal of differential expression in pteridine genes is consistent with a major role of these genes in generating the coloration differences among the three morphs, and is consistent with the limited data available for other amphibians. We found a total of 68 differentially expressed genes linked to pigment production, structural coloration in iridophores, and pigment-cell differentiation which suggests that multiple mechanisms are responsible for the coloration differences, likely involving both pigment-based and structural coloration. In addition to regulatory differences, we found evidence of differential selection acting at the DNA sequence level in several color-associated loci, which underlines the polygenic nature of this color polymorphism. Our research provides an important landmark for future studies on the evolutionary diversification of coloration in *O. pumilio* and other amphibians.

## Methods

### Sampling and sequencing

Field work was conducted in Panama on the Bocas del Toro archipelago from March 25 to March 31 in 2015. We sampled three wild populations of *O. pumilio*: a red population on the mainland at Almirante (N 09°14.706, W 082°22.051), a green population on Isla Popa (N 09°08.500, W 082°07.615) and a blue population on the Tierra Oscura peninsula (N 09°12.685, W 082°12.191). We found large frog populations and captured (between 9:00 and 11:00) five adult males from each locality (15 individuals in total). The frogs were anaesthetized in Tricain S (MS222) and sacrificed by decapitation always at the same time of the day (around 13:00). The dorsal skin was immediately resected and stored in RNAlater. Capture and euthanasia procedures followed ethical guidelines for amphibian research [62] and procedures and export permits were extended by the Panamanian government (ANAM: SC/A-5-15 and SEX/A-25–15) and import permits, of fixed skins, by the Bundesamt für Naturschutz (BafN E-01676/15).

We used the RNeasy Mini Kit (Qiagen) to extract total RNA from the skin samples according to the manufacturers’ instructions, with a final elution in 30 μl. RNA quantity and integrity was measured using a Bioanalyser (Agilent). RNA concentration varied between 106 and 363 ng/μl and the RNA integrity number (RIN) ranged from 8.5 to 9.4. Two μg RNA of each sample were used to create molecularly indexed paired-end cDNA libraries, which were sequenced in 100 bp fragments on an Illumina HiSeq™ 2000 platform. Library preparation and sequencing were performed by BGI (Shenzhen, China).

### Draft genome re-scaffolding

Despite substantial advances in genome assembly algorithms, accurate genome assemblies remain challenging for organisms with large genomes where repetitive regions abound. This challenge is pervasive among amphibians and prominent in the recently assembled *O. pumilio* genome draft, which is a heavily fragmented assembly in spite of extensive and high quality sequence data [63]. In order to re-scaffold the low-quality draft genome of *O. pumilio* we used P_RNA_Scaffolder software [64]. This software, like other recently developed scaffolding algorithms, uses the information from RNA-Seq reads to complete the structures of transcribed regions, since RNA-sequencing captures both mRNAs and long non-coding RNAs [64]. We used as input the paired-end RNA-Seq reads aligned to the original draft genome assembly. RNA-Seq reads obtained in this study derive from skin (15 individuals), liver (2 inds.), and retina (1 ind.). To represent additional tissues, we added the available sequences from brain and ova available at NCBI (SRA accessions: SRR8275033, SRR7639587–90, SRR7639592). Raw Illumina reads were quality-trimmed and adaptor sequences removed using Trimmomatic with default settings. Reads were mapped to the draft genome using HISAT2 [65] and P_RNA_Scaffolder was run on the resulting .bam file using default settings.

### De-novo reference transcriptome assembly

The generation of a reference transcriptome is a crucial step for expression quantification and was challenging in our study as samples from different localities are likely to exhibit substantial variation in expression profiles and probably comprise different genotypes which can result in assembly artifacts [66]. We therefore generated a reference *O. pumilio* transcriptome using a combination of genome-guided and *de-novo* assembly strategies. For the genome-guided approach, we mapped the quality-trimmed reads to the re-scaffolded draft genome of *O. pumilio* [63] using STAR [67] two-pass algorithm with default settings. The mapped reads were then used to generate a genome-guided transcriptome assembly in Trinity [68, 69] with default settings except for maximum intron size = 30 k and minimum k-mer coverage = 2. A de novo transcriptome assembly was also generated using the quality-trimmed reads as input and identical settings in Trinity. Finally, the resulting genome-guided and de-novo transcriptome assemblies were collated into a set of non-redundant transcripts by aligning them to the *O. pumilio* re-scaffolded genome draft using PASA [70]. The PASA pipeline functions applied included: Transcript cleaning (identify and strip polyadenylation, trim vectors, and discard low quality transcripts); mapping and aligning transcripts to the genome (using GMAP [71]); validate nearly perfect alignments (95% identity, over 90% of transcript length, and consensus splice sites); maximal assembly of spliced alignments clustering and assembling of valid transcript alignments based on genome mapping location. To account for the extensive fragmentation of the re-scaffolded genome draft we used the “PASA_comprehensive_db” pipeline to add to the PASA assemblies those *de-novo* or genome-guided assembled transcripts that align partially to the genome but extend onto sequencing gaps (> 30% of transcript length and 95% identity).

The resulting reference transcripts were translated into amino acid sequences using Transdecoder with default settings and retaining transcripts with positive hits against Pfam 32.0 [72] and UniProt [73] databases. Blastp searchers were performed using Diamond [74] with 1 × 10^− 3^ E-value threshold for positive alignments. The blastp hits of the resulting set of coding transcripts were sorted by alignment score, length and E-value and the top-hit blast results were used to annotate the transcripts using Annie [75]. In order to improve functional annotation of transcripts, we searched for orthologs with Uniprot entries using reciprocal best blastp hits (RBBH). We then annotated the resulting list of ortholog transcripts with their corresponding Uniprot RBBH into a transcript-to-gene table. Other transcripts with positive blastp hits against a Uniprot gene already represented in the transcript-to-gene table but residing on a different genomic cluster (i.e, with a different PASA_cluster id) were added to this table but annotated as potential paralogs by adding numeric suffixes to their corresponding gene symbol.

### Differential expression analyses

Transcript abundance in the skin samples was estimated by pseudo-aligning the quality-trimmed reads to the transcriptome reference sequences with Kallisto software [76] using 100 bootstraps for uncertainty estimation. Differential expression analyses were later conducted with the *Sleuth* R-package [77] by comparing the transcript abundance estimates among three groups (blue, red and green morphs) of five individuals each. We imported the transcript abundance estimates of all samples and filtered out all low expressed transcripts, with fewer than five reads in more than 30% of the samples, using a custom filter function in *Sleuth*. We identified differentially expressed (DE) transcripts by comparing a model including color as an explanatory variable against a null model using a likelihood ratio test (LRT). We applied the gene aggregation algorithm in *Sleuth* in which the *p* values of the LRT at the transcript level are weighted by the mean expression level of the transcript and aggregated into genes following a meta-analysis approach. This method outperforms classic “aggregate first and test later” methods where the assignment of a single numerical count value to a gene can mask dynamic effects among its multiple constituent transcripts [78]. We used our transcript-to-gene table, see above, for gene-level aggregation of statistical results and considered as differentially expressed those genes with a false detection ratio (FDR) < 0.05, as calculated from the LRT *p*-values after adjustment for multiple testing with the Benjamini-Hochberg procedure. *Sleuth* calculations were run in R [79]. Batch effects in expression quantification were assumed negligible in our study as all samples were processed as one batch during library preparation and sequencing. An additional inspection of a PCA plot of transcript expression labeled by samples and sequencing lanes (SMFigure 7), revealed no noticeable effect of the sequencing lane.

### Definition of color-associated gene sets

A large number of DE genes are to be expected when comparing expression profiles among samples from the natural populations in this system as genes responsive to any other factor that aligns with coloration will be flagged as DE. The resulting gene lists are then likely to include many genes of which color-associated genes will be a potentially very small fraction. In order to focus on coloration-associated genes, we constructed a list of genes potentially linked to pigment synthesis pathways or structural coloration in previous studies on various vertebrate groups. The resulting list includes 368 genes that have been previously shown to be associated with carotenoid metabolism, pteridine synthesis, melanogenesis, iridophore guanine synthesis, and chromatophore development (SMTable 7). This list is probably not exhaustive as other genes might be associated to coloration in amphibians, and particularly in these frogs, for which the mechanisms of coloration are poorly studied.

Heatmap plots to characterize the expression profiles were drawn using ComplexHeatmaps [80]. Clusters of DE genes were then identified by hierarchical clustering of expression values and functional classification of genes in each subset was performed with the aid of PANTHER [81], at the pathway function level and matching gene symbols against *Xenopus tropicalis* annotations.

### Gene set enrichment and over-representation analyses

In order to further investigate the expression pattern among color phenotypes, we applied Gene-Set Enrichment Analysis (GSEA, [82]) to investigate whether the four color-related gene sets (carotenoid metabolism, pteridine synthesis, melanogenesis, and iridophore guanine synthesis; see SMTable 7), showed differential expression among the three color phenotypes (three groups, five individuals each). GSEA was run for each color pair comparison using the gene-level expression estimates from Kallisto as input and with default settings except for using gene set permutations and minimum size of gene set = 5. Additionally, to characterize the functional significance of the entire set of DE genes we performed a Gene Ontology (GO) term over-representation analysis of these genes using ConsensusPathDB-human [83]. We used the entire set of annotated ortholog genes in the *O. pumilio* transcriptome as background against the over-represented GO-based sets in the DE gene list were searched for. We summarized the resulting long list of enriched gene functional categories by clustering into representative subset of the terms and visualizing cluster importance using a multidimensional plot, as implemented in REVIGO [84].

### SNP detection and quantification of signatures of selection

To overcome the low quality of assembly and annotation of the *O. pumilio* draft genome, we adopted a superTranscriptome approach and used Lace software to combine the assembled transcripts into a set of super-transcripts that contain the sequence of all exons of a gene without redundancy [85]. The gene-to-transcript map, as obtained from PASA, was used as input to Lace to guide the concatenation of transcripts. The resulting superTranscriptome was used as a reference against which all RNA-Seq skin reads of the three color phenotypes were mapped using Hisat2 [65]. Single-Nucleotide Polymorphism (SNP) variants were called using GATK software, following the best-practice workflow for RNA-Seq SNP calling. This pipeline includes: align, sort and mark duplicate read mappings; split reads into exon segments and clip sequences overhanging the intronic regions (SplitNCigarReads); call variants with phred-scaled confidence threshold > 20; filter results based on Fisher Strand values (FS > 30.0) and Qual By Depth values (QD < 2.0). The resulting VCF file was further filtered using vcftools [86] to include only the bi-allelic SNPs, with a minor allele frequency > 0.033 (more than one in 15 diploid individuals), genotyped with >10X coverage and < 50% missing data across individuals. We used the resulting genotypes to identify SNPs showing signatures of selection among the blue, red and green color phenotypes of *O. pumilio* (three groups, five individuals each) using BayeScan software [87]. The required input file was prepared using the R-package radiator (https://github.com/thierrygosselin/radiator). BayeScan was run for 100,000 iterations (50,000 discarded as burn-in) and sampled every 10th iteration, with a prior odds value of 10 and a false discovery rate (FDR) of 0.05. Twenty pilot runs were used to choose the proposal distribution for the reversible jump MCMC algorithm, convergence of the run was assessed from the values of effective sizes and visual inspection of the traces of the sampled output using the coda R-package. We mapped the PASA comprehensive collection of transcripts to super-transcriptome using GMAP [71] and predicted the functional impact of the outlier SNPs on the transcript structures using SNPdat [88].

## Supplementary information

**Additional file 1 SMTable 1:** Assembly statistics of the original *Oophaga pumilio* draft genome and the re-scaffolded draft produced for this study.

**Additional file 2 SMTable 2:** Results of the differential expression analysis between blue, green and red color phenotypes of *O. pumilio*, obtained using *Sleuth* software. For each gene, the number of transcripts compared, q-value, expression pattern and color-associated function are presented. Putative paralogs are denoted in the symbol column by a “_” followed by a number.

**Additional file 3 SMTable 3:** Results of the functional classification of gene expression clusters at the pathway level. The results were obtained in PANTHER using the symbols of the differentially expressed genes mapped against the *Xenopus tropicalis* annotation.

**Additional file 4 SMTable 4:** Results of the over-representation analysis of the 1445 genes found to be differentially expressed between color phenotypes of *O. pumilio* frogs. Analysis was conducted using ConsensusPathDB. GO category names whose hypergeometric *p*-value < 0.01 are listed, and for each set, the set size (the absolute size, as well as the corrected size), the over-representation p-value, the q-value (false discovery rate), the set size and all input genes in each set are provided.

**Additional file 5 SM Table 5:** Annotated SNPs showing signatures of positive selection in BayeScan analyses. Putative paralogs are denoted in the symbol column by a “_” followed by a number. Functional impact of SNPs was evaluated on the transcripts of each gene using SNPdat. SNPs on genes that also showed significant expression differences are highlighted in bold (see main text for further details).

**Additional file 6 SM Table 6:** Differentially expressed genes in skin samples of different color phenotypes of *Oophaga pumilio* and *Dendrobates auratus* [33].

**Additional file 7 SM Table 7:** List of the color-associated genes, compiled from the literature and used for functional interpretation.

**Additional file 8 Supplementary figures.**

## Data Availability

The raw sequencing reads have been deposited in NCBI Sequence Read Archive (under bioproject: PRJNA610154). Additional reads from previous studies, used to assemble the reference transcriptome, are available at NCBI (SRA accessions: SRR8275033, SRR7639587–90, SRR7639592). The newly assembled comprehensive transcriptome of *Oophaga pumilio* is available in the Transcriptome Shotgun Assembly (TSA) database (accession: GIKS00000000). The re-scaffolded genome and the super transcriptome of *Oophaga pumilio* obtained in this study are available at Zenodo [89]. The datasets supporting the conclusions of this article are included within the article and eight additional files.

## Abbreviations

*adcy6*: Adenylate cyclase type 6
*adsl*: Adenylosuccinate lyase
*aldh1a1*: Retinal dehydrogenase 1
*atic*: Bifunctional purine biosynthesis protein PURH
*atp12a*: Potassium-transporting ATPase alpha chain 2
*bco2*: Beta,beta-carotene 9′,10′-oxygenase
BUSCO: Benchmarking universal single-copy ortholog genes
*camk2g*: Calcium/calmodulin-dependent protein kinase type II subunit gamma
CDS: Coding sequences
*ctnnb1*: Catenin beta-1
CYP: Cytochrome P 450 family genes
*cyp8b1*: 5-beta-cholestane-3-alpha,7-alpha-diol 12-alpha-hydroxylase
DE: Diferencially expressed
*dgat2*: Diacylglycerol O-acyltransferase 2
*dhrs3*: Short-chain dehydrogenase/reductase 3
*dock7*: Dedicator of cytokinesis protein 7
*eno3*: Beta-enolase
*ep300*: Histone acetyltransferase p300
*fh*: Fumarate hydratase, mitochondrial
*gart*: Trifunctional purine biosynthetic protein adenosine-3
*gnai1*: Guanine nucleotide-binding protein G(i) subunit alpha-1
GO: Gene Ontology term
GTP: Guanosine triphosphate
*hps1*: Hermansky-Pudlak syndrome 1 protein homolog
*hps3*: Hermansky-Pudlak syndrome 3 protein
*hps4*: Hermansky-Pudlak syndrome 4 protein
*mdh1a*: Malate dehydrogenase, cytoplasmic
*med1*: Mediator of RNA polymerase II transcription subunit 1
*med12*: Mediator of RNA polymerase II transcription subunit 12
*myo5a*: Unconventional myosin-Va
*oca2*: P protein
*paics*: Multifunctional protein ADE2
*pax7*: Paired box protein Pax-7
*pck2*: Phosphoenolpyruvate carboxykinase [GTP], mitochondrial
*pfas*: Phosphoribosylformylglycinamidine synthase
*phgdh*: D-3-phosphoglycerate dehydrogenase
*plcb4*: 1-phosphatidylinositol 4,5-bisphosphate phosphodiesterase beta-4
*ppat*: Amidophosphoribosyltransferase
PRPP: 5-Phosphoribosyl 1-Pyrophosphate
*prtfdc1*: Phosphoribosyltransferase domain-containing protein 1
*psat1*: Phosphoserine aminotransferase
*pts*: 6-pyruvoyl tetrahydrobiopterin synthase
*raf1*: RAF proto-oncogene serine/threonine-protein kinase
*rdh10*: Retinol dehydrogenase 10
*rdh16*: Retinol dehydrogenase 16
RNA: Ribonucleic acid
SAICAR: phosphoribosylaminoimidazole-succinocarboxamide
*scarb1*: Scavenger receptor class B member 1
*sf3b1*: Splicing factor 3B subunit 1
*shmt2*: Serine hydroxymethyltransferase, mitochondrial
SNP: Single nucleotide polymorphism
*sox7*: Transcription factor Sox-7
*spr*: Sepiapterin reductase
UTR: Untranslated regions
*wnt10b*: Protein Wnt-10b
*wnt9a*: Protein Wnt-9a
*xdh*: Xanthine dehydrogenase/oxidase
