# Supplementary material for: Being red, blue and green: the genetic basis of coloration differences in the strawberry poison frog (Oophaga pumilio)
Source: BMC Genomics. 2020 Apr 15;21:301. doi: 10.1186/s12864-020-6719-5 (PMC7158012; doi:10.1186/s12864-020-6719-5)
Supplement: Supplementary file 1 — Additional file 1 SMTable 1: Assembly statistics of the original Oophaga pumilio draft genome and the re-scaffolded draft produced for this study. [file 12864_2020_6719_MOESM1_ESM.docx]

| Assembly | 1st Draft  (Rogers et al. 2018) | re-scaffolded Draft  (this study) |
| --- | --- | --- |
| # contigs (>= 0 bp) | 7,182,834 | 631,034 |
| # contigs (>= 1000 bp) | 180,920 | 166,613 |
| # contigs (>= 5000 bp) | 97,273 | 81,478 |
| # contigs (>= 10000 bp) | 80,348 | 66,113 |
| # contigs (>= 25000 bp) | 50,762 | 41,342 |
| # contigs (>= 50000 bp) | 28,580 | 24,887 |
| Total length (>= 0 bp) | 5,569,294,449 | 4,837,165,062 |
| Total length (>= 1000 bp) | 4,644,407,837 | 4,662,030,311 |
| Total length (>= 5000 bp) | 4,473,167,038 | 4,491,606,290 |
| Total length (>= 10000 bp) | 4,355,796,610 | 4,385,515,797 |
| Total length (>= 25000 bp) | 3,865,905,421 | 3,978,460,778 |
| Total length (>= 50000 bp) | 3,067,690,657 | 3,389,673,868 |
| # contigs | 290,011 | 271,872 |
| Largest contig | 966,297 | 1,737,582 |
| Total length | 4,721,340,152 | 4,736,188,396 |
| GC (%) | 43 | 43 |
| N50 | 75,719 | 108,981 |
| N75 | 35,027 | 42,717 |
| L50 | 17,079 | 10,963 |
| L75 | 39,842 | 28,403 |
| # N's per 100 kbp | 24,379 | 24,528 |
| Complete BUSCO (%) | 77 | 85 |
| Partial BUSCO (%) | 15 | 7 |

SMTable 1. Assembly statistics of the original *Oophaga pumilio* draft genome and the re-scaffolded draft produced for this study.
