# Supplementary material for: Being red, blue and green: the genetic basis of coloration differences in the strawberry poison frog (Oophaga pumilio)
Source: BMC Genomics. 2020 Apr 15;21:301. doi: 10.1186/s12864-020-6719-5 (PMC7158012; doi:10.1186/s12864-020-6719-5)
Supplement: Supplementary file 8 — Additional file 8 Supplementary figures. [file 12864_2020_6719_MOESM8_ESM.docx]

Supplemental figures for:

**Being red, blue and green: the genetic basis of coloration differences in the strawberry poison frog (*Oophaga pumilio*)**

Ariel Rodríguez^1*^, Nicholas I. Mundy^2^, Roberto Ibáñez^3,4^ and Heike Pröhl^1^

^1^ Institute of Zoology, University of Veterinary Medicine of Hannover, Bünteweg 17, 30559 Hannover

[ariel.rodriguez@tiho-hannover.de](mailto:ariel.rodriguez@tiho-hannover.de); [heike.proehl@tiho-hannover.de](mailto:heike.proehl@tiho-hannover.de)

^2^ Department of Zoology, University of Cambridge, [Downing St](http://www.zoo.cam.ac.uk), [Cambridge](http://www.zoo.cam.ac.uk) , [CB2 3EJ](http://www.zoo.cam.ac.uk)

[nim21@cam.ac.uk](mailto:nim21@cam.ac.uk)

^3^ Smithsonian Tropical Research Institute, Apartado Postal 0843-03092, Panamá, República de Panamá

[ibanezr@si.edu](mailto:ibanezr@si.edu)

^4^ Sistema Nacional de Investigación, Secretaría Nacional de Ciencia, Tecnología e Innovación, Apartado 0816-02852, Panamá, República de Panamá


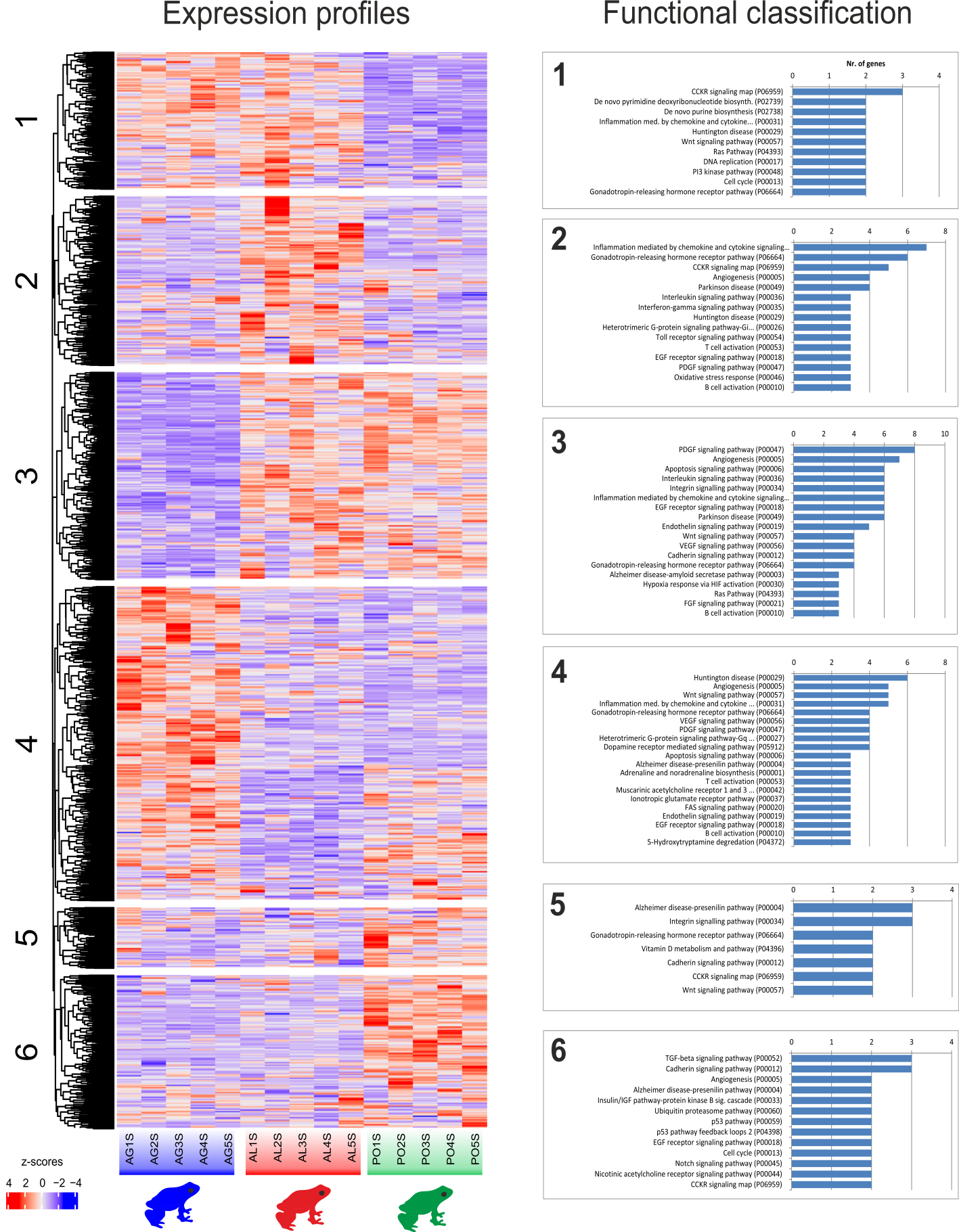


SMFigure 1. Expression profiles and functional classification of genes differentially expressed in skin of three color phenotypes of *Oophaga pumilio* frogs. The six main gene clusters are numbered and their corresponding functional classification, at the pathway level, is summarized with bar charts on the right side. Differential expression at the gene level was performed with *Sleuth* and functional classification of gene sets with PANTHER. Only the main pathways for each cluster are plotted (see SMTable X for the full details).


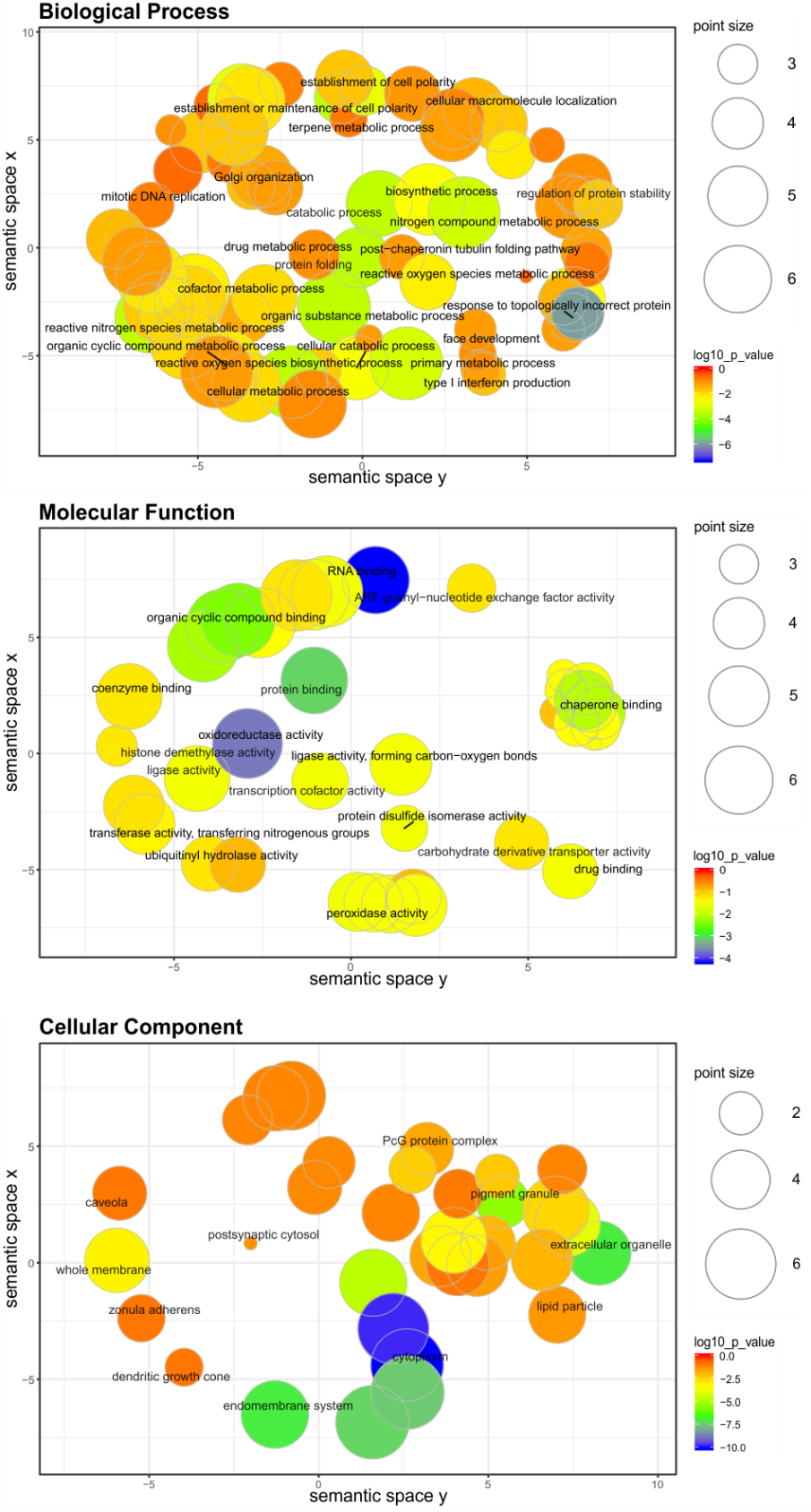


SMFigure 2. Scatterplot of the two main axes obtained after a multidimensional scaling of a matrix of the semantic similarities calculated from the GO terms in each category. Only the cluster representatives (i.e. terms remaining after the redundancy reduction) are labeled. Bubble color indicates the q-value, obtained in the over-representation analysis, and size indicates the frequency of the GO term in the underlying GOA database (UNIPROT) (bubbles of more general terms are larger). The plots were rendered using REVIGO.


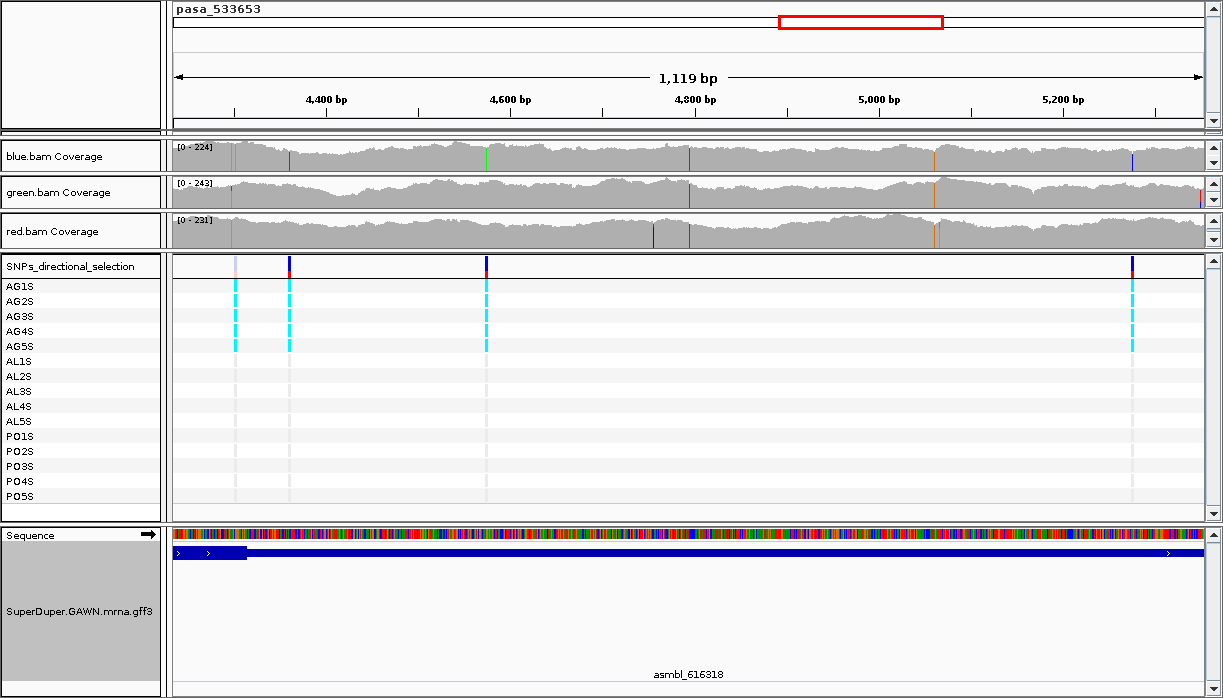


SMFigure 3. Integrative Genomics Viewer plot showing the location of the four SNPs with strong signature of diversifying selection in the super-transcript of the *kit* gene. The upper three panels show the RNA-Seq reads mapping coverage of the three color phenotypes, the middle panel shows the four outlier SNPs, detected by BayeScan, and the lower panel a collapsed view of the transcript structures in this supertranscript region.


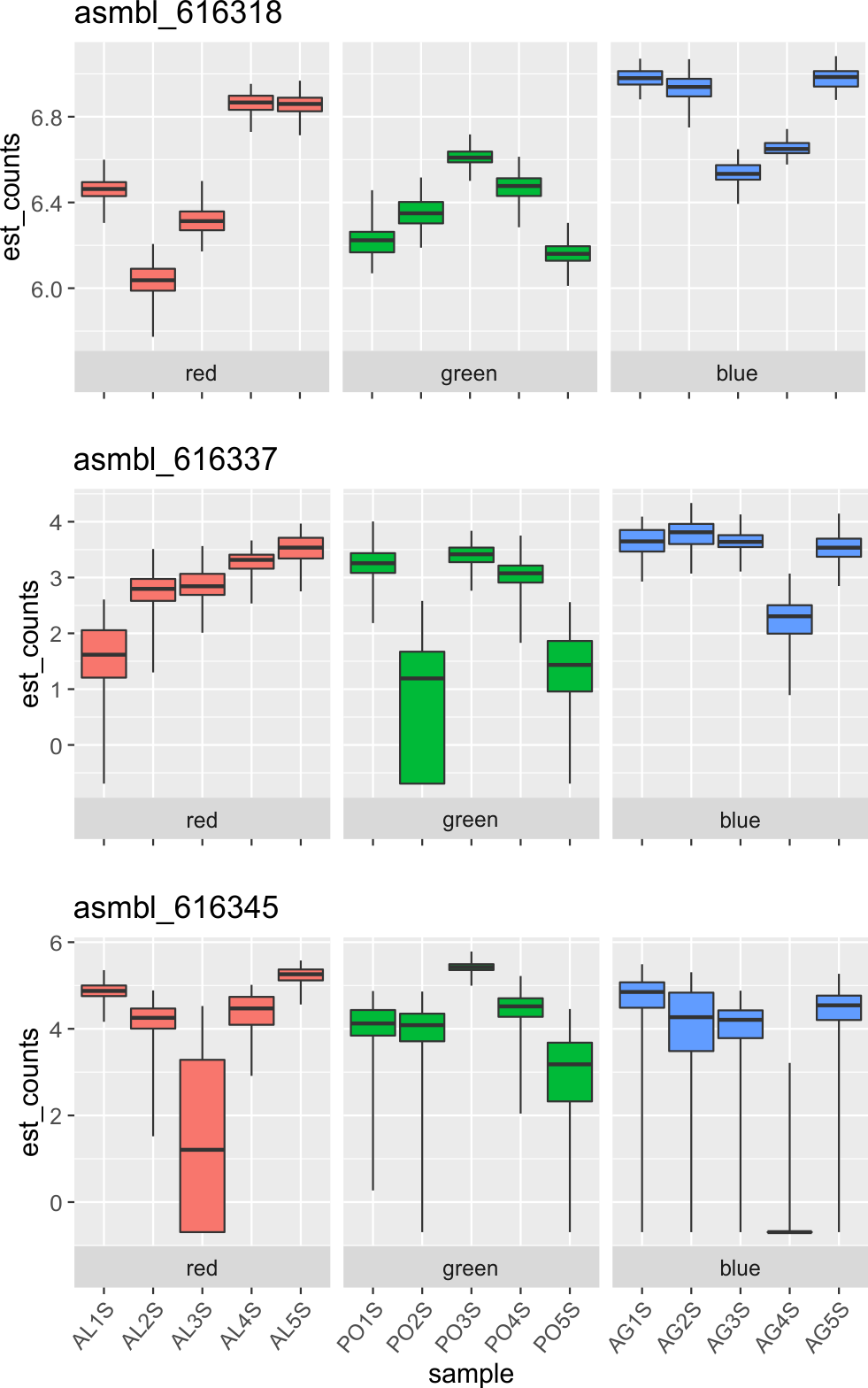


SMFigure 4. Expression profiles of the three transcripts of the *kit* gene quantified using *Sleuth* for each sample of the three color phenotypes of *Oophaga pumilio*. For each transcript the box-plots summarize the read counts estimated with bootstrapping.


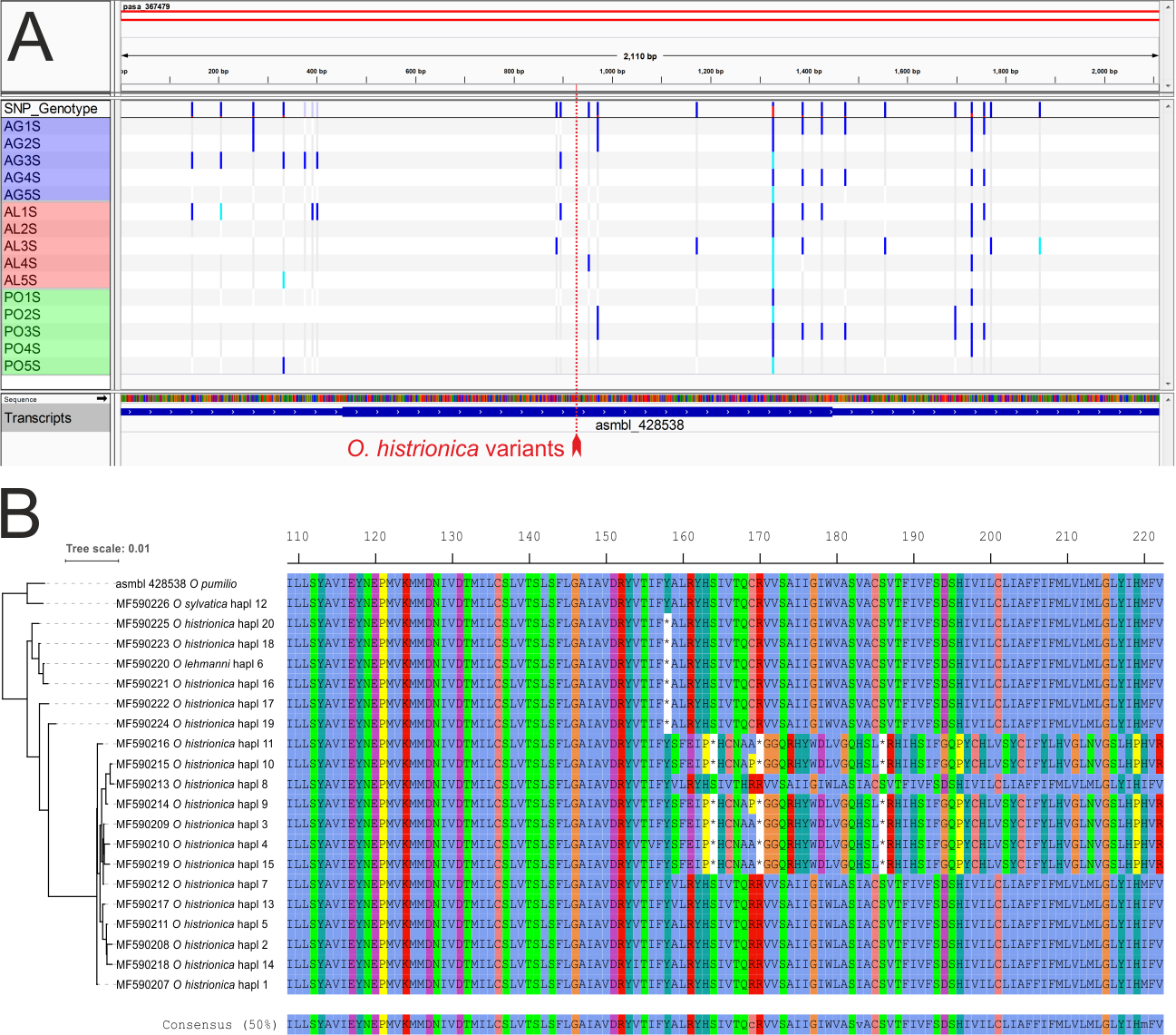


SMFigure 5. Variation in the MC1R locus is not associated to the blue, red or green color phenotypes of *Oophaga pumilio*. A) IGV plot displaying the 22 SNP genotype variants identified in the MC1R gene. Samples are coded as in figure 1 and genotypes are color-coded as: dark blue = heterozygous, cyan = homozygous variant, grey = reference. The lower blue track represents the transcript structure (thin bands: UTR regions, thick band: CDS). The vertical red arrow indicates the location of previously published variants associated with dorsal coloration in *O. histrionica* and *O. lehmanni*, no variants were detected in this region in *O. pumilio*. B) Multiple sequence alignment of the protein sequences encoded by the MC1R locus in *Oophaga pumilio* (top row in alignment) and other *Oophaga* frogs. The asterisks indicate the stop codons leading to truncated receptor structures previously associated with darker skin coloration in some *O. histrionica* and *O. lehmanni* populations but absent in *O. sylvatica* and the three *O. pumilio* populations studied.


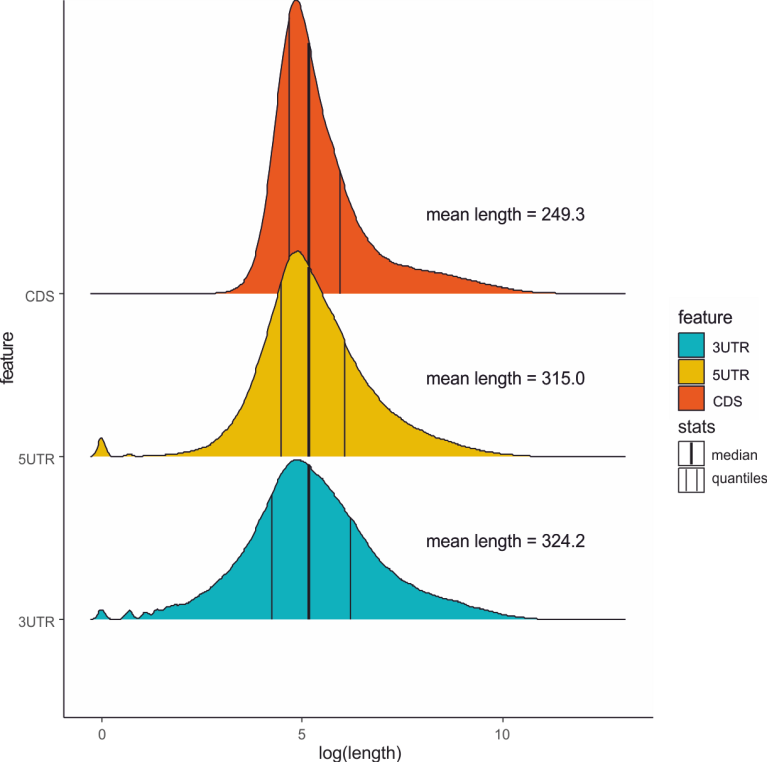


SMFigure 6. Distribution of feature lengths in the reference transcriptome of *Oophaga pumilio*.


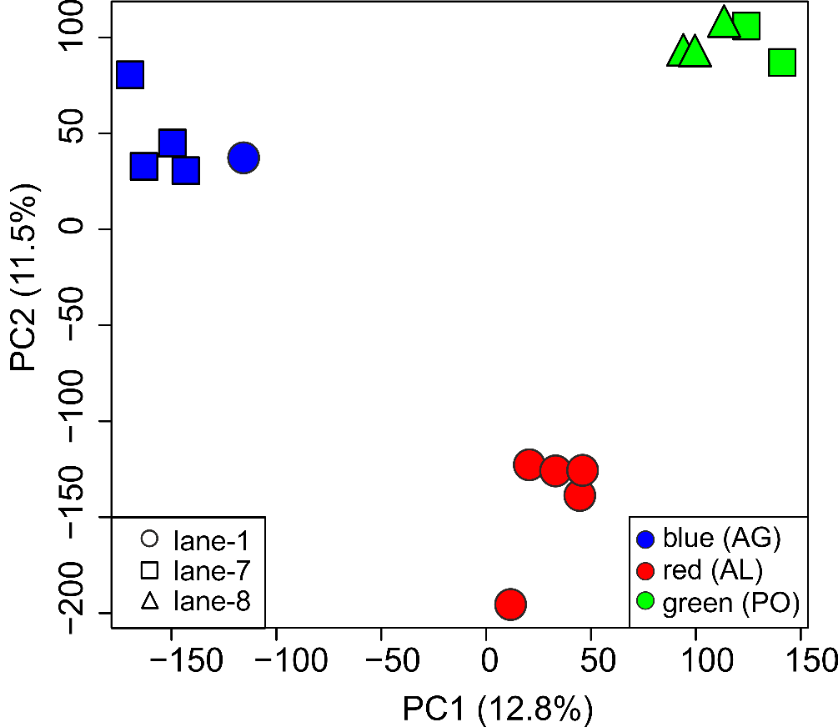


SMFigure 7. Plot of the principal component analysis summarizing the expression pattern across samples of *Oophaga pumilio* showing three color phenotypes (AL, Almirante; AG, Aguacate; PO, Popa) with symbols identifying the original sequencing lanes.
